# Effect of Empagliflozin on Worsening Heart Failure Events in Patients With Heart Failure and Preserved Ejection Fraction: EMPEROR-Preserved Trial

**DOI:** 10.1161/CIRCULATIONAHA.121.056824

**Published:** 2021-08-29

**Authors:** Milton Packer, Javed Butler, Faiez Zannad, Gerasimos Filippatos, Joao Pedro Ferreira, Stuart J. Pocock, Peter Carson, Inder Anand, Wolfram Doehner, Markus Haass, Michel Komajda, Alan Miller, Steen Pehrson, John R. Teerlink, Sven Schnaidt, Cordula Zeller, Janet M. Schnee, Stefan D. Anker

**Affiliations:** Baylor Heart and Vascular Institute, Baylor University Medical Center, Dallas, TX (M.P.).; Imperial College, London, United Kingdom (M.P.).; Department of Medicine, University of Mississippi School of Medicine, Jackson (J.B.).; Université de Lorraine, Inserm INI-CRCT, CHRU, Nancy, France (F.Z., J.P.F.).; National and Kapodistrian University of Athens School of Medicine, Athens University Hospital Attikon, Greece (G.F.).; Cardiovascular Research and Development Center, Department of Surgery and Physiology, Faculty of Medicine of the University of Porto, Portugal (J.P.F.).; Department of Medical Statistics, London School of Hygiene and Tropical Medicine, United Kingdom (S.J.P.).; Washington DC Veterans Affairs Medical Center (P.C.).; Department of Cardiology, University of Minnesota, Minneapolis (I.A.).; Department of Cardiology (CVK) and Berlin Institute of Health Center for Regenerative Therapies, German Centre for Cardiovascular Research Partner Site Berlin, Charité Universitätsmedizin, Berlin, Germany (W.D., S.D.A.).; Theresienkrankenhaus and St Hedwig-Klinik, Mannheim, Germany (M.H.).; Department of Cardiology, Hospital Saint Joseph, Paris, France (M.K.).; University of Florida, Jacksonville (A.M.).; Department of Cardiology, University Hospital, Rigshospitalet, Copenhagen, Denmark (S.P.).; Section of Cardiology, San Francisco Veterans Affairs Medical Center and School of Medicine, University of California (J.R.T.).; Biostatistics and Data Sciences, Boehringer Ingelheim Pharma GmbH & Co KG, Biberach, Germany (S.S., C.Z.).; Boehringer Ingelheim Pharmaceuticals, Inc, Ridgefield, CT (J.M.S.).

**Keywords:** heart failure, sodium-glucose transporter 2 inhibitors

## Abstract

**Background:**

Empagliflozin reduces the risk of cardiovascular death or hospitalization for heart failure in patients with heart failure with preserved ejection fraction, but additional data are needed about its effect on inpatient and outpatient heart failure events.

**Methods:**

We randomly assigned 5988 patients with class II through IV heart failure with an ejection fraction of >40% to double-blind treatment with placebo or empagliflozin (10 mg once daily), in addition to usual therapy, for a median of 26 months. We prospectively collected information on inpatient and outpatient events reflecting worsening heart failure and prespecified their analysis in individual and composite end points.

**Results:**

Empagliflozin reduced the combined risk of cardiovascular death, hospitalization for heart failure, or an emergency or urgent heart failure visit requiring intravenous treatment (432 versus 546 patients [empagliflozin versus placebo, respectively]; hazard ratio, 0.77 [95% CI, 0.67–0.87]; *P*<0.0001). This benefit reached statistical significance at 18 days after randomization. Empagliflozin reduced the total number of heart failure hospitalizations that required intensive care (hazard ratio, 0.71 [95% CI, 0.52–0.96]; *P*=0.028) and the total number of all hospitalizations that required a vasopressor or positive inotropic drug (hazard ratio, 0.73 [95% CI, 0.55–0.97]; *P*=0.033). Compared with patients in the placebo group, fewer patients in the empagliflozin group reported outpatient intensification of diuretics (482 versus 610; hazard ratio, 0.76 [95% CI, 0.67–0.86]; *P*<0.0001), and patients assigned to empagliflozin were 20% to 50% more likely to have a better New York Heart Association functional class, with significant effects at 12 weeks that were maintained for at least 2 years. The benefit on total heart failure hospitalizations was similar in patients with an ejection fraction of >40% to <50% and 50% to <60%, but was attenuated at higher ejection fractions.

**Conclusions:**

In patients with heart failure with preserved ejection fraction, empagliflozin produced a meaningful, early, and sustained reduction in the risk and severity of a broad range of inpatient and outpatient worsening heart failure events.

**Registration:**

URL: https://www.clinicaltrials.gov; Unique identifier: NCT03057977.

## Abstract 2

Supplemental Digital Content is available in the text.

**Clinical Perspective**

**What Is New?**

- EMPEROR-Preserved (Empagliflozin Outcome Trial in Patients With Chronic Heart Failure With Preserved Ejection Fraction) showed that, in patients with heart failure and preserved ejection fraction, empagliflozin reduced the primary end point of cardiovascular death or hospitalization for heart failure, primarily related to a 29% lower risk of hospitalizations for heart failure.
- In the current analysis, we show that empagliflozin reduced the risk of severe hospitalizations, as reflected by admissions requiring the use of positive inotropic and vasopressor drugs and the need for intensive care.
- Empagliflozin also reduced the risk of outpatient worsening heart failure events, including need for urgent care visits, diuretic intensification, and unfavorable changes in functional class.

**What Are the Clinical Implications?**

- Therapeutic options for patients with heart failure and preserved ejection fraction are limited; previous trials with various interventions have shown little or modest effects.
- The favorable effects of empagliflozin on inpatient and outpatient worsening heart failure events in patients with heart failure and preserved ejection fraction in EMPEROR-Preserved are similar to those reported with empagliflozin in patients with heart failure and reduced ejection fraction in EMPEROR-Reduced (Empagliflozin Outcome Trial in Patients With Chronic Heart Failure With Reduced Ejection Fraction).

Heart failure is a progressive disorder marked by ongoing or episodic worsening of symptoms, leading to a deterioration of functional capacity and requiring intensification of treatment. Often, these episodes of clinical worsening can be managed in an outpatient setting, which can involve an office, clinic, emergency department, or urgent care setting. When severe or of rapid onset, worsening symptoms may require hospitalization, and if the patient becomes clinically unstable, admission to intensive care. Therefore, the clinical progression of heart failure can manifest itself in a broad range of clinical settings. Although the primary end point of clinical trials typically focuses only on hospitalizations,^1,2^ these represent only a small proportion of a patient’s journey,^3,4^ and outpatient and inpatient worsening heart failure events can have similar adverse prognostic implications.^5–7^

In clinical trials in type 2 diabetes or chronic kidney disease, sodium-glucose cotransporter 2 (SGLT2) inhibitors reduced the risk of hospitalizations for heart failure.^8–14^ However, these trials did not evaluate the effect of these drugs on the broad spectrum of both inpatient and outpatient heart failure events. We and others have reported that in patients with heart failure with reduced ejection fraction, empagliflozin, dapagliflozin, and sotagliflozin not only lowered the risk of hospitalization for heart failure, but also diminished the risk of outpatient events, including emergency or urgent care visits and outpatient intensification of diuretics for worsening heart failure.^15–18^ In this article, we report on the effect of empagliflozin on inpatient and outpatient worsening heart failure events in patients with heart failure and an ejection fraction >40%. Furthermore, because patients with heart failure with preserved ejection fraction (HFpEF) have comorbidities that can cause serious disability for reasons other than heart failure, we report on the effect of empagliflozin using more comprehensive definitions of death and hospitalization.^19–23^

## Methods

EMPEROR-Preserved (Empagliflozin Outcome Trial in Patients With Chronic Heart Failure With Preserved Ejection Fraction; URL: https://www.clinicaltrials.gov; Unique identifier: NCT0305791) was a randomized, double-blind, parallel-group, placebo-controlled, event-driven study, whose design has been described previously.^24,25^ Ethics approval was obtained at each study site and informed consent was obtained from all study participants. Data will be made available on request in adherence with transparency conventions in medical research and through requests to the corresponding author. The Executive Committee of the EMPEROR trials (Empagliflozin Outcome Trial in Patients With Chronic Heart Failure) has developed a comprehensive analysis plan and numerous prespecified analyses, which will be presented in future scientific meetings and publications. At a later time point, the full database will be made available in adherence with the transparency policy of the sponsor (available at https://trials.boehringer-ingelheim.com/transparency_policy.html).

### Study Patients and Assessments

We enrolled men or women with chronic heart failure (New York Heart Association [NYHA] functional class II, III, or IV) with a left ventricular ejection fraction >40% who had elevated levels of NT-proBNP (N-terminal prohormone B-type natriuretic peptide) >300 pg/mL; this threshold was tripled in patients with atrial fibrillation at baseline. Patients who fulfilled prespecified inclusion and exclusion criteria were randomized double-blind (in a 1:1 ratio) to receive placebo or empagliflozin 10 mg daily in addition to their usual therapy. After randomization, all appropriate treatments for heart failure or other medical conditions could be initiated or altered at the discretion of the physician of each patient. Patients were assessed at study visits for major outcomes, functional capacity related to heart failure, changes in the use of diuretics, vital signs, and biomarkers reflecting changes in the course of heart failure or the action of SGLT2 inhibitors; these assessments took place every 2 to 6 months, depending on the metric and the duration of follow-up. All randomized patients were to be followed for prespecified outcomes for the entire duration of the trial.

### Trial End Points

In a manner similar to that followed for EMPEROR-Reduced (Empagliflozin Outcome Trial in Patients With Chronic Heart Failure With Reduced Ejection Fraction),^15^ we prospectively collected information on deaths, hospitalizations for any reason, and emergency, urgent, and outpatient events that reflect worsening heart failure. Hospitalizations were classified as cardiovascular or noncardiovascular on the basis of the judgment of the investigator, but hospitalizations for heart failure were prospectively adjudicated by a clinical event committee in a blinded manner using prespecified criteria. To qualify as an adjudicated heart failure hospitalization, patients were required to have meaningful worsening of their clinical status and intensification of treatment for heart failure. The duration of the in-hospital stay was at least 12 hours; if the patient had not received intravenous medications for heart failure, the minimum stay of an adjudicated heart failure hospitalization was 24 hours. Investigators documented the clinical course of each hospital admission on a dedicated form, including the prescribing of medications used to treat episodes of clinical decompensation and the use of intensive care.

In addition to the adjudication and characterization of hospitalizations for heart failure, at each scheduled study visit, patients were prospectively asked about interval events and about changes in the use of diuretics that were prescribed in response to worsening heart failure since the most recent visit. Events that were prospectively considered to reflect meaningful changes in clinical status included (1) worsening heart failure that required the use of an intravenous drug for heart failure in an emergency department or urgent care setting; (2) intensification of daily doses of diuretics for worsening symptoms; and (3) changes in NYHA functional class. Events treated in an emergency department, urgent care setting, or during a hospital stay shorter than that required for an adjudicated event were grouped together. Physicians were not provided any specific guidance as to the degree or duration of diuretic intensification that would qualify as representing a clinically meaningful change. Ascertainment of these events and measures were prospectively collected in the case report form and their inclusion in analyses of individual and composite end points was prespecified before the blind of the trial was broken. Outpatient worsening heart failure events were not adjudicated.

The end points selected for inclusion in this article are similar to those that we analyzed for EMPEROR-Reduced,^15^ and we prospectively planned to perform these analyses for EMPEROR-Preserved.

### Statistical Analysis

For time-to-first-event analyses, differences between the placebo and empagliflozin groups were assessed using a Cox proportional hazards model, with prespecified covariates of age, sex, geographical region, diabetes status at baseline, left ventricular ejection fraction, and estimated glomerular filtration rate at baseline. To determine the time point when statistical significance was reached and maintained for the first time, the same Cox regression models were fitted and sequentially censored at increasing number of days since randomization, yielding a continuous display of hazard ratios (HRs) with confidence bands. For the analysis of total (first and repeated) events, between-group differences were assessed using a joint frailty model,^26^ with cardiovascular death (for end points including heart failure events) or all-cause mortality (for end points including all-cause hospitalization) as competing risks and using the covariates that were used for the time-to-first-event analyses.

Odds ratios were calculated for the effect of empagliflozin versus placebo on NYHA class at prespecified study visits using ordinal logistic regression assuming partial proportional odds adjusting for baseline NYHA class and using the same covariates as for the Cox regression models, without imputation for missing data. For the analysis of changes in vital signs and biomarkers, treatment effects were assessed on the basis of changes from baseline using a mixed model for repeated measures that included age and baseline estimated glomerular filtration rate and ejection fraction as linear covariates and baseline score by visit, visit by treatment, sex, region, individual last projected visit on the basis of dates of randomization and trial closure, and baseline diabetes status as fixed effects. The analysis of changes in N-terminal proBNP was performed on log-transformed data. Because the outcome measures were highly correlated, no adjustment was made for multiplicity of comparisons.

## Results

A total of 5988 patients were randomly assigned to placebo (n=2991) or to empagliflozin (n=2997). As previously reported,^25^ the 2 groups had clinical features typical of patients with HFpEF and they were well-balanced with respect to baseline characteristics.

### Effect on Combined Risk of Death or Hospitalization

There were 662 patients who died for any reason or were hospitalized for heart failure in the placebo group and 581 such patients in the empagliflozin group, reflecting a 15% lower risk with empagliflozin compared with placebo (HR, 0.85 [95% CI, 0.76–0.95]; *P*=0.005; Table 1). There were 967 patients who died for any reason or were hospitalized for a cardiovascular reason in the placebo group and 888 such patients in the empagliflozin group, reflecting a 11% lower risk with empagliflozin than placebo (HR, 0.89 [95% CI, 0.81–0.98]; *P*=0.014). There were 1431 patients who died or were hospitalized for any reason in the placebo group and 1356 such patients in the empagliflozin group, reflecting an 8% lower risk with empagliflozin than placebo (HR, 0.92 [95% CI, 0.85–0.99]; *P*=0.025; Table 1).

**Table 1.**
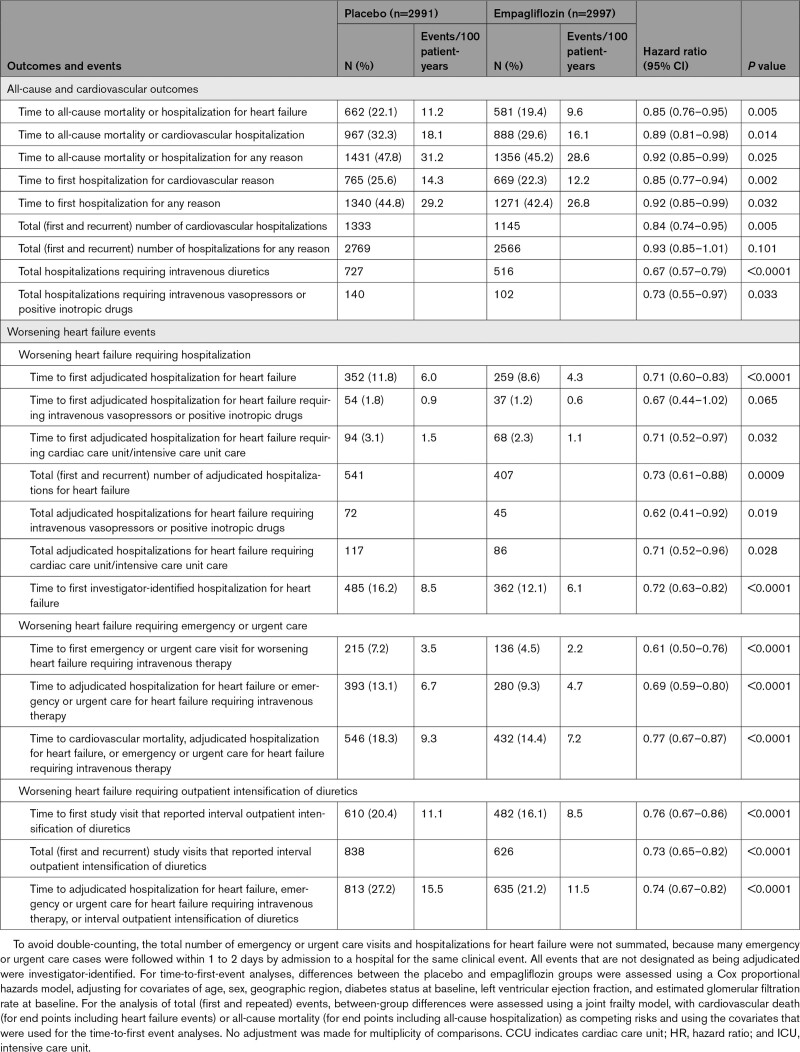
Major Outcomes and Worsening Heart Failure Events

### Effect of Empagliflozin on Hospitalizations

**Figure 1.**
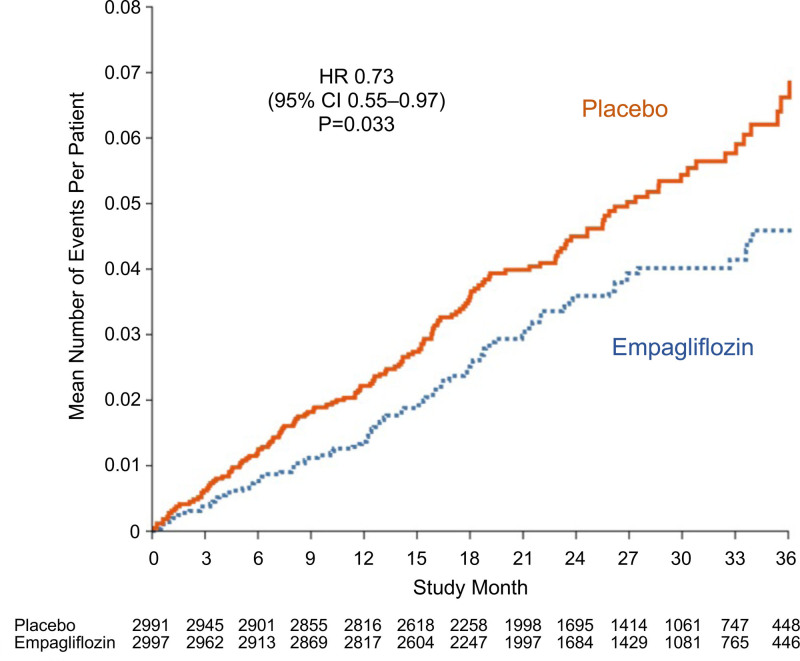
**Total (first and recurrent) hospitalizations for any reason that required intravenous vasopressors or positive inotropic agents.** Shown are mean cumulative function curves for placebo (shown in red) and for empagliflozin (shown in blue). HR indicates hazard ratio.

Compared with the placebo group, patients in the empagliflozin group had fewer total (first and recurrent) hospitalizations for heart failure (407 versus 541; HR, 0.73 [95% CI, 0.61–0.88]; *P*=0.0009) and fewer total (first and recurrent) hospitalizations for a cardiovascular reason (1145 versus 1333; HR, 0.84 [95% CI, 0.74–0.95]; *P*=0.005). Total (first and recurrent) hospitalizations for any reason were not significantly different in the 2 treatment groups (2566 versus 2769; empagliflozin versus placebo, respectively; HR, 0.93 [95% CI, 0.85–1.01]; *P*=0.10; Table 1). However, among these, empagliflozin reduced the total number of hospitalizations requiring intravenous diuretics by 33% (HR, 0.67 [95% CI, 0.57–0.79]; *P*<0.0001) and the total number of hospitalizations requiring intravenous vasopressor or positive inotropic agents by 27% (HR, 0.73 [95% CI, 0.55–0.97]; *P*=0.03; Figure 1).

The effect of empagliflozin on total hospitalizations for heart failure is described in Table 2. Compared with patients in the placebo group, fewer patients in the empagliflozin group were hospitalized for heart failure once (181 versus 243), hospitalized for heart failure twice (49 versus 67), and hospitalized for heart failure 3 or more times (29 versus 42). The effect of empagliflozin on total (first and recurrent) hospitalizations for heart failure was consistent in most of the prespecified subgroups. For this end point, we observed a nominally significant interaction between treatment and the baseline use of mineralocorticoid receptor antagonists (interaction *P*=0.038) as well as an interaction between treatment and ejection fraction (*P* trend=0.008), with an attenuated response in patients with an ejection fraction ≥60% (Figure I in the Data Supplement). When the analysis of hospitalizations was broadened to total (first and recurrent) cardiovascular hospitalizations, only the interaction between treatment and ejection fraction was still apparent (*P* trend=0.02), again with an attenuated response in patients with an ejection fraction ≥60% (Figure II in the Data Supplement).

**Table 2.**
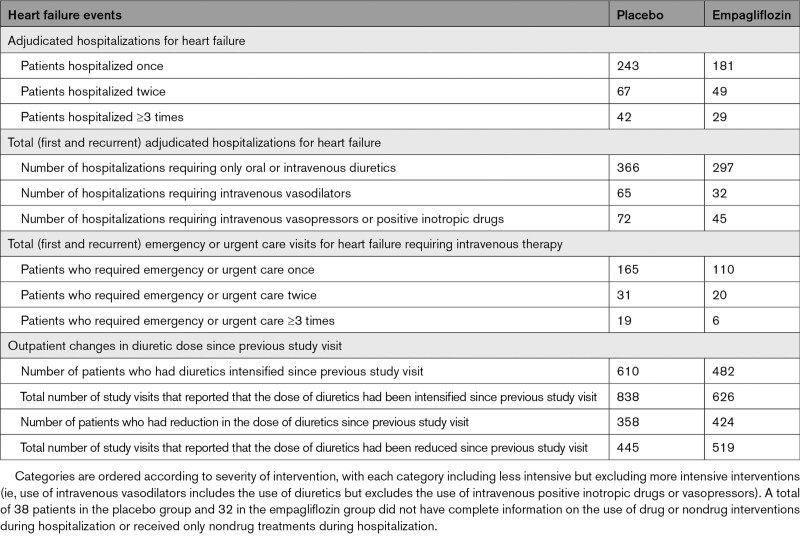
Occurrence of Worsening Heart Failure Events

**Figure 2.**
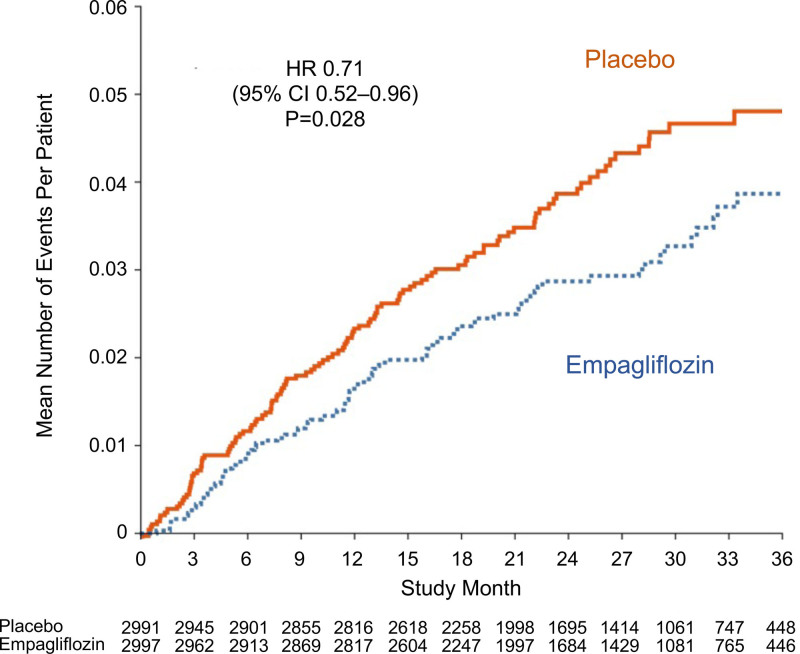
**Total (first and recurrent) adjudicated heart failure hospitalizations requiring admission to cardiac care unit or intensive care unit in the placebo and empagliflozin groups.** Shown are mean cumulative function curves for placebo (shown in red) and for empagliflozin (shown in blue). HR indicates hazard ratio.

Empagliflozin also reduced the severity of heart failure admissions and the frequency of use of a broad range of interventions used for the management of decompensated heart failure. Empagliflozin prolonged the time to the first heart failure hospitalization that required intensive care and decreased the total number of such heart failure admissions (HR, 0.71 [95% CI, 0.52–0.96]; *P*=0.03; Table 1 and Figure 2). When compared with the placebo group, the empagliflozin group experienced fewer total hospitalizations for heart failure that required only oral or intravenous diuretics (297 versus 366), fewer hospitalizations for heart failure that required intravenous vasodilators but no vasopressor or positive inotropic agents (32 versus 65), and fewer admissions for heart failure that required a vasopressor or positive inotropic agent (45 versus 72; Table 2). When considering all hospitalizations for heart failure, the 2 groups were similar with respect to the mean duration of each admission for heart failure (10.8 [95% CI, 9.6–12.0] and 11.5 [95% CI, 10.0–12.9] for placebo versus empagliflozin, respectively; *P*=0.50).

### Effect of Empagliflozin on Worsening Heart Failure Events Other Than Hospitalizations

**Figure 3.**
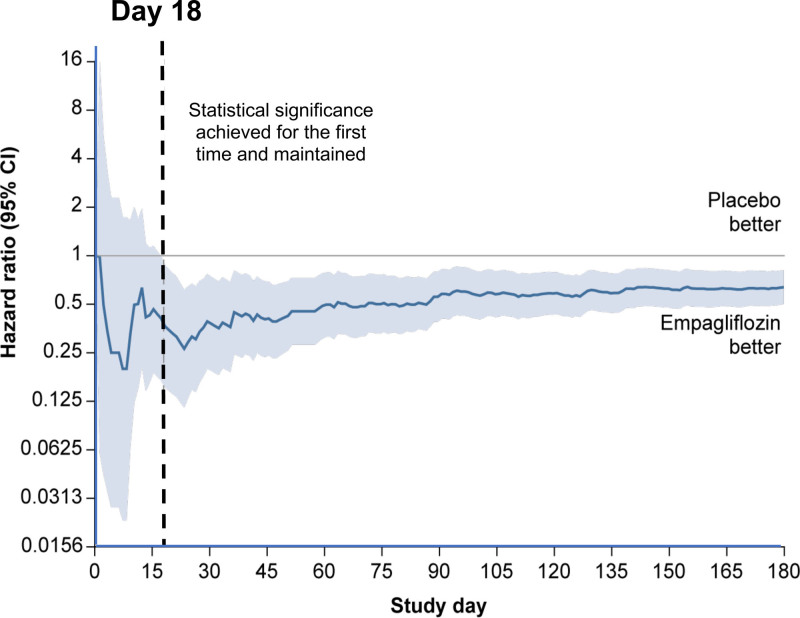
**Time of first statistical significance in time-to-first-event analysis of cardiovascular death, hospitalization for heart failure, or emergency or urgent heart failure visit requiring intravenous treatment for worsening heart failure.** To determine the time point when statistical significance was reached and maintained for the first time, Cox regression models were fitted and sequentially censored at increasing number of days since randomization, yielding a continuous display of hazard ratios with confidence bands.

Patients in the empagliflozin group experienced fewer emergency or urgent care visits for worsening heart failure (298 events in the placebo group and 174 in the empagliflozin group; HR, 0.55 [95% CI, 0.43–0.69]; *P*<0.0001). When a worsening heart failure event is defined as cardiovascular death, hospitalization for heart failure, or an emergency or urgent heart failure visit requiring intravenous treatment, there were 546 events in the placebo group and 432 events in the empagliflozin group, reflecting a 23% lower risk of a worsening heart failure event with empagliflozin (HR, 0.77 [95% CI, 0.67–0.87]; *P*<0.0001; Table 1). The benefit of empagliflozin on this end point first reached statistical significance at 18 days after randomization and maintained significance thereafter (Figure 3).

**Figure 4.**
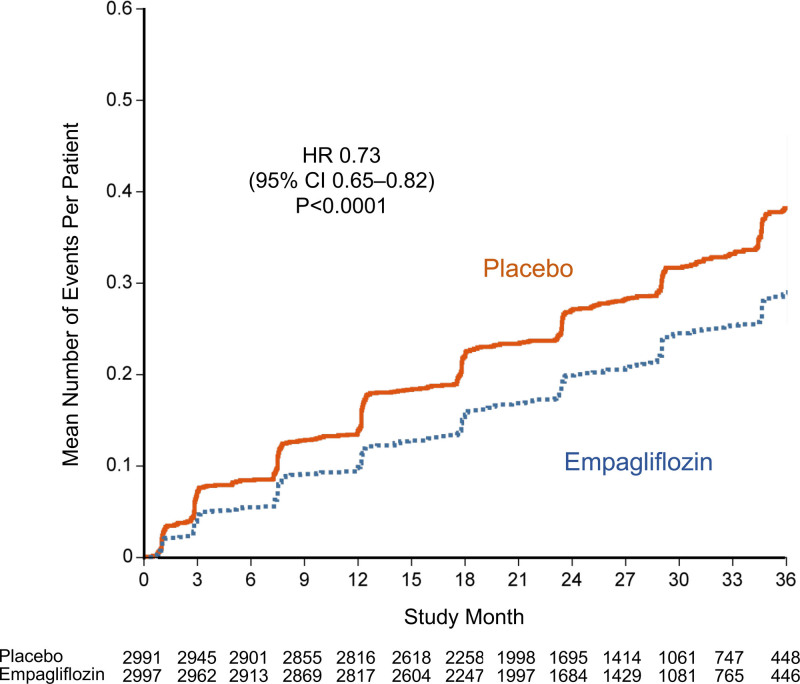
**Total number of outpatient visits reporting interval intensification of diuretics for worsening heart failure.** Shown are mean cumulative function curves for placebo (shown in red) and for empagliflozin (shown in blue). HR indicates hazard ratio.

Compared with placebo, fewer patients in the empagliflozin group reported outpatient intensification of diuretics since the prior study visit (482 versus 610) and there were fewer total study visits that reported interval outpatient diuretic intensification in the empagliflozin group (626 versus 838; Tables 1 and 2). Conversely, the empagliflozin group had more patients (424 versus 358) and more study visits (519 versus 445) where the dose of diuretics was reported to have been reduced. Empagliflozin prolonged the time to the first study visit that reported interval outpatient diuretic intensification (HR, 0.76 [95% CI, 0.67-0.86]; *P*<0.0001, and the drug reduced the total number of study visits that reported interval outpatient intensification of diuretics since the prior visit (HR, 0.73 [95% CI, 0.65–0.82]; *P*<0.0001; Table 1 and Figure 4).

In general, at each prespecified study visit, patients in the empagliflozin group were at 20% to 50% greater odds of having a less severe NYHA functional class than patients in the placebo group (Table 3). This treatment difference was nearly significant at week 4 (*P*=0.06) and was statistically significant at all time points from 12 weeks through 148 weeks after randomization (Table 3).

**Table 3.**
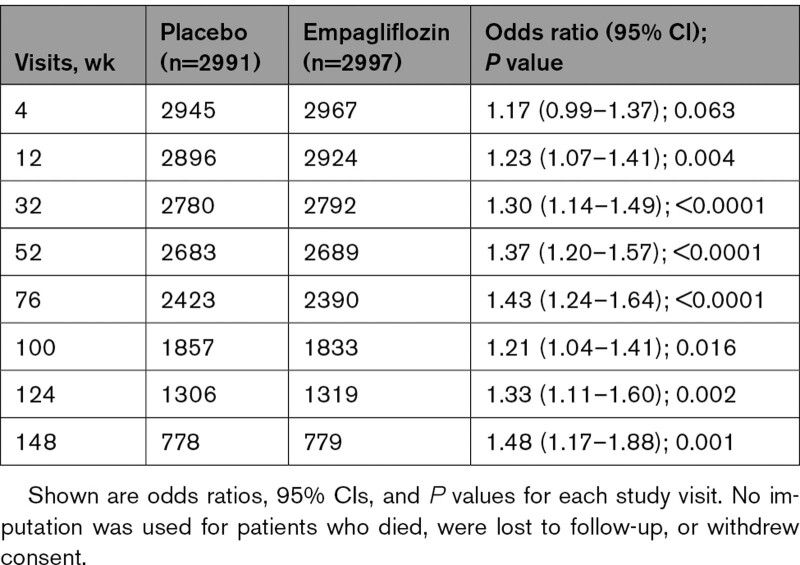
Odds Ratios (Empagliflozin: Placebo) for a Less Severe New York Heart Association Functional Class at Planned Study Visits (Partial Proportional Odds Model), Without Imputation

Serial changes in laboratory tests and vital signs are shown in Figures III through VII in the Data Supplement. Treatment with empagliflozin was accompanied by small decreases in NT-proBNP after 4 weeks and the effect increased in magnitude over time. Empagliflozin produced significant increases in hematocrit and decreases in uric acid, which were apparent as early as 4 weeks after randomization and were maintained for >24 months. Patients treated with empagliflozin experienced an early and sustained decrease in body weight and systolic blood pressure, which averaged somewhat greater than 1 kg and 2 mm Hg, respectively.

## Discussion

As previously reported, in EMPEROR-Preserved, empagliflozin reduced the risk of cardiovascular death or hospitalization for heart failure by 21% (*P*<0.001), an effect that was related primarily to reduction in hospitalizations for heart failure.^25^ We show that when the analysis is expanded to include all deaths and all hospitalizations regardless of attribution, the effect of empagliflozin on morbidity and mortality remained statistically significant (*P*=0.025). However, as expected, the magnitude of the effect on hospitalization diminished when the focus of the analysis broadened beyond heart failure events, decreasing from a 27% risk reduction for total heart failure hospitalizations to 16% risk reduction for total cardiovascular hospitalizations to 7% risk reduction for total hospitalizations for any reason. This decline is expected whenever hospitalizations that are not particularly influenced by a treatment are added in a stepwise manner to the analysis of events in a trial of patients with HFpEF, in whom only 46% of the hospital admissions are related to a cardiovascular reason and only 18% are related to worsening heart failure.

Empagliflozin reduced the risk of hospitalization for heart failure, whether the analysis was broadened to investigator-identified events or focused on adjudicated events. The hospitalizations that were prevented by treatment with empagliflozin involved admissions that were treated only with oral or intravenous diuretics as well as hospitalizations that required therapy with intravenous vasopressors or positive inotropic agents or that necessitated intensive care. These benefits of SGLT2 inhibition were seen in time-to-first-event analyses and in the analysis of total (first and recurrent) events; the number of patients who were hospitalized once or multiple times was fewer in the empagliflozin group than in the placebo group. Empagliflozin reduced the total number of hospitalizations for heart failure that involved admission to intensive care by 29% (HR, 0.71 [95% CI, 0.52–0.96]) and the total number of admissions for any reason that required intravenous vasopressors or positive inotropic drugs by 27% (HR, 0.73 [95% CI, 0.55–0.97]). The lower risk of hospital admissions did not result in a longer length of stay when patients in the empagliflozin group were hospitalized for heart failure.

In addition to these inpatient events, treatment with empagliflozin had an important effect to mitigate worsening heart failure events in the outpatient setting. There were fewer emergency or urgent care visits for worsening heart failure in the empagliflozin group than in the placebo group. SGLT2 inhibition reduced the number of study visits that reported diuretic intensification for worsening heart failure by 25%, and at the same time, empagliflozin led to a 17% increase in the number of study visits that reported a decrease in dose of diuretics. Moreover, patients in the empagliflozin group had a 20% to 50% higher odds of having a better NYHA functional class; these benefits were apparent early in treatment and were sustained for the duration of double-blind therapy. An early symptom effect is concordant with our finding on clinical events; that is, when worsening heart failure was defined in a manner similar to the primary end point of DAPA-HF (Study to Evaluate the Effect of Dapagliflozin on the Incidence of Worsening Heart Failure or Cardiovascular Death in Patients With Chronic Heart Failure; eg, cardiovascular death, hospitalization for heart failure, or an emergency or urgent heart failure visit requiring intravenous treatment),^27^ a benefit of empagliflozin was apparent (HR, 0.77 [95% CI, 0.67–0.87]) and first reached statistical significance at 18 days after randomization and remained significant for the duration of follow-up. This finding of early benefits is similar to that which we and others have reported with SGLT2 inhibitors in patients with heart failure with reduced ejection fraction.^15,27^

The effects of empagliflozin on inpatient and outpatient worsening heart failure events was largely consistent across our predefined subgroups. We noted a possible influence of concomitant treatment with mineralocorticoid receptor antagonists in an analysis of the effect of empagliflozin on total heart failure hospitalizations, but this interaction was no longer apparent when we broadened the analysis to all cardiovascular hospitalizations. In contrast, ejection fraction influenced the magnitude of the effect of empagliflozin when the analysis focused on total hospitalizations for heart failure (*P* trend=0.008) or on all cardiovascular hospitalizations (*P* trend=0.02). These findings are concordant with our yet-to-be-published observations that the effect of empagliflozin to reduce the risk of heart failure hospitalization is similar in magnitude across a broad range of ejection fractions ranging from <25% to <65% but is attenuated in patients with an ejection fraction of 65% or greater. Further characterization of patients with heart failure and high-normal ejection fractions is warranted.

The mechanisms by which empagliflozin reduces inpatient and outpatient worsening heart failure events are not well understood. Although the early benefits of empagliflozin to prevent clinical deterioration may suggest the possibility of a natriuretic effect,^28^ the magnitude of such an action is typically modest and short-lived.^29–31^ Conventional diuretics typically produce immediate declines in natriuretic peptides, generally without a change in hematocrit. In contrast, the early effect of empagliflozin on natriuretic peptides was very small in our trial, and hematocrit increased (presumably as a result of erythrocytosis^32^), a pattern similar to that seen with empagliflozin in patients with heart failure with reduced ejection fraction.^15^ Many patients with HFpEF are hypertensive, and systolic blood pressure decreased modestly with empagliflozin. Sacubitril/valsartan produces greater decreases in blood pressure, but more modest decreases in the risk of hospitalization and emergency or urgent care visits.^33–35^ Obesity can play an important role in HFpEF,^36,37^ but the decline in body weight with empagliflozin was too small to mitigate the influence of adiposity. Inflammation and oxidative stress have been implicated in the pathogenesis of HFpEF,^38,39^ and empagliflozin produced meaningful and sustained decreases in uric acid, a marker of oxidative stress.^40^ SGLT2 inhibitors have been shown to ameliorate inflammation and oxidative stress in experimental HFpEF.^41,42^ The importance of these mechanisms in the clinical setting remain to be fully explored.

The findings of the present study should be interpreted in light of its strengths and limitations. This trial is the largest randomized double-blind controlled trial in patients with HFpEF, and we prospectively collected information on inpatient and outpatient worsening heart failure events; the outpatient clinical course of these patients has not been well-characterized to date. However, noninvasive assessment of cardiac structure and function were not standardized or interpreted in a central laboratory. Thus, we were not able to explore the heterogeneity of this patient population, and our measurements of ejection fraction are subject to the variability typically seen in clinical practice.

In conclusion, in patients with HFpEF, SGLT2 inhibition with empagliflozin produced a meaningful, early, and sustained reduction in the risk and severity of a broad range of inpatient and outpatient worsening heart failure events. These benefits included a decrease in the need for hospitalizations requiring aggressive therapy, a diminution of worsening events requiring intensification of diuretics, and an increased likelihood of functional class improvement, effects that were maintained for >2 years of double-blind treatment.

## Sources of Funding

Boehringer Ingelheim and Eli Lilly and Company.

## Disclosures

Dr Packer reports receiving consulting fees from Boehringer Ingelheim during the conduct of the study and consulting fees from AbbVie, Actavis, Amgen, Amarin, AstraZeneca, Bristol Myers Squibb, Casana, CSL Behring, Cytokinetics, Johnson & Johnson, Lilly, Moderna, Novartis, ParatusRx, Pfizer, Relypsa, Salamandra, Synthetic Biologics, and Theravance, outside the submitted work. Dr Zannad has received steering committee or advisory board fees from Amgen, AstraZeneca, Bayer, Boehringer Ingelheim, Boston Scientific, Cardior, CVRx, Janssen, Livanova, Merck, Mundipharma, Novartis, Novo Nordisk, and Vifor Fresenius and personal fees from Boehringer Ingelheim during the conduct of the study. Dr Butler reports receiving consulting fees from Boehringer Ingelheim, Cardior, CVRx, Foundry, G3 Pharma, Imbria, Impulse Dynamics, Innolife, Janssen, LivaNova, Luitpold, Medtronic, Merck, Novartis, NovoNordisk, Relypsa, Roche, Sanofi, Sequana Medical, V-Wave Ltd, and Vifor and personal fees from Boehringer Ingelheim during the conduct of the study. Dr Fillipatos reports committee member contributions in trials and personal fees from Boehringer Ingelheim during the conduct of the study. Dr Ferreira is a consultant for Boehringer Ingelheim. Dr Pocock is a consultant for Boehringer Ingelheim and received personal fees from Boehringer Ingelheim during the conduct of the study. Dr Carson received consulting fees from Boehringer Ingelheim and IQVIA related to work on a clinical events committee during the conduct of the study. Dr Anand reports receiving consulting fees from Boehringer Ingelheim and IQVIA related to work on a clinical events committee during the conduct of the study and personal/consulting fees from ARCA, Amgen, Boston Scientific Corporation, Novartis, LivaNova, and Zensun. Dr Doehner reports receiving consulting fees from Boehringer Ingelheim related to work on a clinical events committee during the conduct of the study; personal fees from Aimediq, Bayer, Boehringer Ingelheim, Medtronic, Pfizer, Sanofi-Aventis, Sphingotec, and Vifor Pharma; and research support from EU (Horizon2020), German ministry of Education and Research, German Center for Cardiovascular Research, Vifor Pharma, and ZS Pharma. Dr Haass received consulting fees from Boehringer Ingelheim related to work on a clinical events committee during the conduct of the study. Dr Komajda received consulting fees from Boehringer Ingelheim related to work on a clinical events committee during the conduct of the study and personal fees from Novartis, Servier, Amgen, Sanofi, Bayer, AstraZeneca, Lilly, and Torrent. Dr Miller reports receiving consulting fees from Abbott, Boehringer Ingelheim, Respicardia, CVRx, Pfizer, and AbbVie. Dr Pehrson reports receiving consulting fees and/or lecture fees from Boehringer Ingelheim, Glaxo Smith Kline, Celgene, Bristol Myers Squibb, Bayer, and Johnson & Johnson. Dr Teerlink reports receiving grants and/or consulting fees from Abbott, Amgen, Astra-Zeneca, Bayer, Boehringer-Ingelheim, Cytokinetics, Daxor, EBR Systems, LivaNova, Medtronic, Merck, Novartis, Relypsa, Servier, Windtree Therapeutics, and ZS Pharma. S. Schnaidt, C. Zeller, and Dr Schnee are employees of Boehringer Ingelheim. Dr Anker reports grants and personal fees from Vifor Int and Abbott Vascular; personal fees from Astra-Zeneca, Bayer, Brahms, Boehringer Ingelheim, Cardiac Dimensions, Novartis, Occlutech, Servier, and Vifor Int; and personal fees from Boehringer Ingelheim during the conduct of the study.

## Supplemental Materials

Data Supplement Figures I–VII

## Supplementary Material


