# Supplementary material for: Effect of Empagliflozin on Worsening Heart Failure Events in Patients With Heart Failure and Preserved Ejection Fraction: EMPEROR-Preserved Trial
Source: Circulation. 2021 Aug 29;144(16):1284–94. doi: 10.1161/CIRCULATIONAHA.121.056824 (PMC8522627; doi:10.1161/CIRCULATIONAHA.121.056824)
Supplement: Supplementary file 1 [file cir-144-1284-s001.pdf]

SUPPLEMENTAL MATERIAL

Packer et al., Effect of Empagliflozin on Worsening Heart Failure Events in Patients with Heart Failure and a Preserved Ejection Fraction: the EMPEROR-Preserved Trial

Supplemental Figure I
Total (first and recurrent) hospitalizations for worsening heart failure, by subgroups

Supplemental Figure II
Total cardiovascular hospitalizations, by subgroups

Supplemental Figure III
Changes in hematocrit during double-blind treatment in the placebo and empagliflozin groups

Supplemental Figure IV
Changes in uric acid during double-blind treatment in the placebo and empagliflozin groups

Supplemental Figure V
Changes in body weight during double-blind treatment in the placebo and empagliflozin groups

Supplemental Figure VI
Changes in N-terminal proBNP during double-blind treatment in the placebo and empagliflozin groups

Supplemental Figure VII
Changes in systolic blood pressure during double-blind treatment in the placebo and empagliflozin groups

<u>Supplemental Figure I</u>

Total (first and recurrent) hospitalizations for worsening heart failure, by subgroups

Subgroup	Empagliflozin	Placebo	Hazard ratio (95% CI)	Interaction *p*-value
	no. of events / no. of patients			
Overall	407/2997	541/2991	0.73 (0.61–0.88)	
Baseline diabetes status				0.9697
Diabetes	250/1466	335/1472	0.73 (0.57–0.94)	
No diabetes	157/1531	206/1519	0.74 (0.56–0.97)	
Age				0.0832
<70 yr	141/1066	159/1084	0.92 (0.67–1.26)	
≥70 yr	266/1931	382/1907	0.65 (0.52–0.82)	
Sex				0.7800
Male	253/1659	308/1653	0.75 (0.59–0.95)	
Female	154/1338	233/1338	0.71 (0.53–0.94)	
Race				0.4276
White	282/2286	368/2256	0.74 (0.59–0.92)	
Black	27/133	21/125	1.30 (0.55–3.11)	
Asian	76/413	119/411	0.58 (0.37–0.91)	
Other	22/164	33/198	0.79 (0.37–1.69)	
Baseline body-mass index				0.0749
<30	211/1654	312/1642	0.63 (0.49–0.81)	
≥30	196/1343	229/1349	0.88 (0.67–1.15)	
Baseline eGFR (CKD-EPI)				0.1369
≥60 mL/min/1.73 m²	150/1493	178/1505	0.88 (0.66–1.18)	
<60 mL/min/1.73 m²	257/1504	363/1484	0.66 (0.52–0.85)	
Baseline systolic blood pressure				0.9094
<Median	215/1496	290/1474	0.74 (0.57–0.95)	
≥Median	192/1501	251/1517	0.72 (0.55–0.94)	
History of atrial fibrillation or atrial flutter				0.9424
No	164/1417	223/1427	0.72 (0.54–0.95)	
Yes	243/1576	318/1559	0.73 (0.57–0.94)	
Baseline LVEF (%)				0.0077
<50	122/995	209/988	0.57 (0.42–0.79)	
≥50 to <60	129/1028	182/1030	0.66 (0.48–0.91)	
≥60	156/974	150/973	1.06 (0.76–1.46)	
Heart failure hospitalization in ≤12 mo				0.6868
No	200/2298	271/2321	0.70 (0.56–0.88)	
Yes	207/699	270/670	0.76 (0.56–1.04)	
Baseline NYHA class				0.2899
II	235/2435	347/2452	0.67 (0.54–0.83)	
III/IV	172/562	194/539	0.84 (0.59–1.19)	
Baseline NT-proBNP (calculated by atrial fibrillation/flutter status)				0.7560
<Median	104/1477	152/1508	0.69 (0.51–0.94)	
≥Median	303/1516	388/1476	0.73 (0.58–0.93)	
Baseline uric acid (tertiles)				0.6194
<T1	92/963	131/975	0.67 (0.47–0.95)	
≥T1 and <T2	99/1032	124/966	0.82 (0.58–1.16)	
≥T2	213/970	281/1018	0.75 (0.56–0.99)	
Baseline use of ACE-inhibitor, ARB or ARNI				0.8612
No	117/569	151/587	0.71 (0.48–1.04)	
Yes	290/2428	390/2404	0.74 (0.60–0.91)	
Baseline use of MRA				0.0379
No	201/1878	308/1866	0.60 (0.47–0.77)	
Yes	206/1119	233/1125	0.90 (0.68–1.19)	

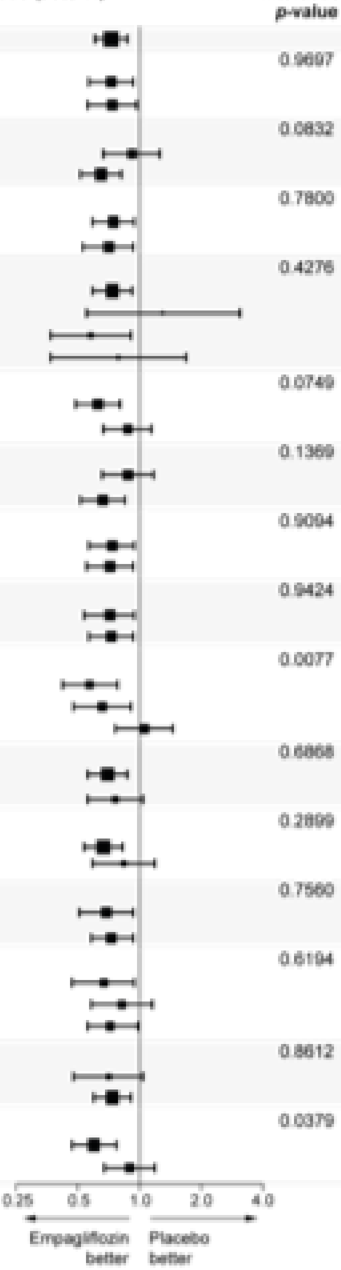

Trend test p-value is shown for baseline uric acid (using sex-specific tertiles, T1 and T2) and ejection fraction subgroups. The size of the squares for the hazard ratios is proportional to the size of the subgroup. The body-mass index is the weight in kilograms divided by the square of the height in meters. Race was reported by the patients. NYHA class II includes 4 patients with NYHA class I. Baseline uric acid tertiles calculated separately for males and females. ARNi= angiotensin receptor–neprilysin inhibitor, CKD-EPI= Chronic Kidney Disease Epidemiology Collaboration, HF= heart failure, eGFR= estimated glomerular filtration rate, LVEF= left ventricular ejection fraction, MRA= mineralocorticoid receptor antagonist, NT-proBNP= N-terminal prohormone of brain natriuretic peptide, NYHA= New York Heart Association.

<u>**Supplemental Figure II**</u>
Total cardiovascular hospitalizations, by subgroups

Subgroup	Empagliflozin	Placebo	Hazard ratio (95% CI)	Interaction p-value
	no. of events / no. of patients			
Overall	1145/2997	1333/2991	0.84 (0.74–0.95)	
Baseline diabetes status				0.3444
Diabetes	616/1466	777/1472	0.79 (0.67–0.94)	
No diabetes	529/1531	556/1519	0.89 (0.75–1.07)	
Age				0.6384
<70 yr	374/1066	442/1084	0.87 (0.71–1.07)	
≥70 yr	771/1931	891/1907	0.82 (0.71–0.95)	
Sex				0.5625
Male	675/1659	751/1653	0.86 (0.73–1.02)	
Female	470/1338	582/1338	0.80 (0.67–0.97)	
Race				0.3763
White	865/2286	975/2256	0.86 (0.75–0.99)	
Black	68/133	72/125	0.97 (0.56–1.71)	
Asian	146/413	211/411	0.65 (0.47–0.90)	
Other	66/164	75/198	0.98 (0.59–1.63)	
Baseline body-mass index				0.0589
<30	594/1654	740/1642	0.75 (0.64–0.89)	
≥30	551/1343	593/1349	0.95 (0.80–1.14)	
Baseline eGFR (CKD-EPI)				0.4614
≥60 mL/min/1.73 m²	426/1493	535/1505	0.80 (0.67–0.96)	
<60 mL/min/1.73 m²	719/1504	798/1484	0.88 (0.75–1.04)	
Baseline systolic blood pressure				0.5002
<Median	583/1496	658/1474	0.87 (0.73–1.04)	
≥Median	562/1501	675/1517	0.80 (0.68–0.95)	
History of atrial fibrillation or atrial flutter				0.7213
No	480/1417	592/1427	0.81 (0.68–0.98)	
Yes	665/1576	741/1559	0.85 (0.72–1.00)	
Baseline LVEF (%)				0.0176
<50	324/995	462/988	0.69 (0.56–0.86)	
≥50 to <60	416/1028	471/1030	0.85 (0.69–1.04)	
≥60	405/974	400/973	1.00 (0.81–1.24)	
Heart failure hospitalization in ≤12 mo				0.3209
No	723/2298	810/2321	0.88 (0.76–1.01)	
Yes	422/699	523/670	0.77 (0.61–0.96)	
Baseline NYHA class				0.2299
II	756/2435	942/2452	0.79 (0.69–0.91)	
III/IV	389/562	391/539	0.94 (0.73–1.21)	
Baseline NT-proBNP (calculated by atrial fibrillation/flutter status)				0.9428
<Median	428/1477	499/1508	0.84 (0.70–1.00)	
≥Median	717/1516	833/1476	0.83 (0.71–0.98)	
Baseline uric acid (tertiles)				0.2854
<T1	279/963	365/975	0.72 (0.58–0.91)	
≥T1 and <T2	339/1032	372/966	0.93 (0.75–1.16)	
≥T2	514/970	588/1018	0.86 (0.70–1.05)	
Baseline use of ACE-inhibitor, ARB or ARNI				0.7135
No	295/569	309/587	0.87 (0.67–1.14)	
Yes	850/2428	1024/2404	0.83 (0.72–0.95)	
Baseline use of MRA				0.7320
No	697/1878	822/1866	0.82 (0.70–0.96)	
Yes	448/1119	511/1125	0.86 (0.71–1.05)	

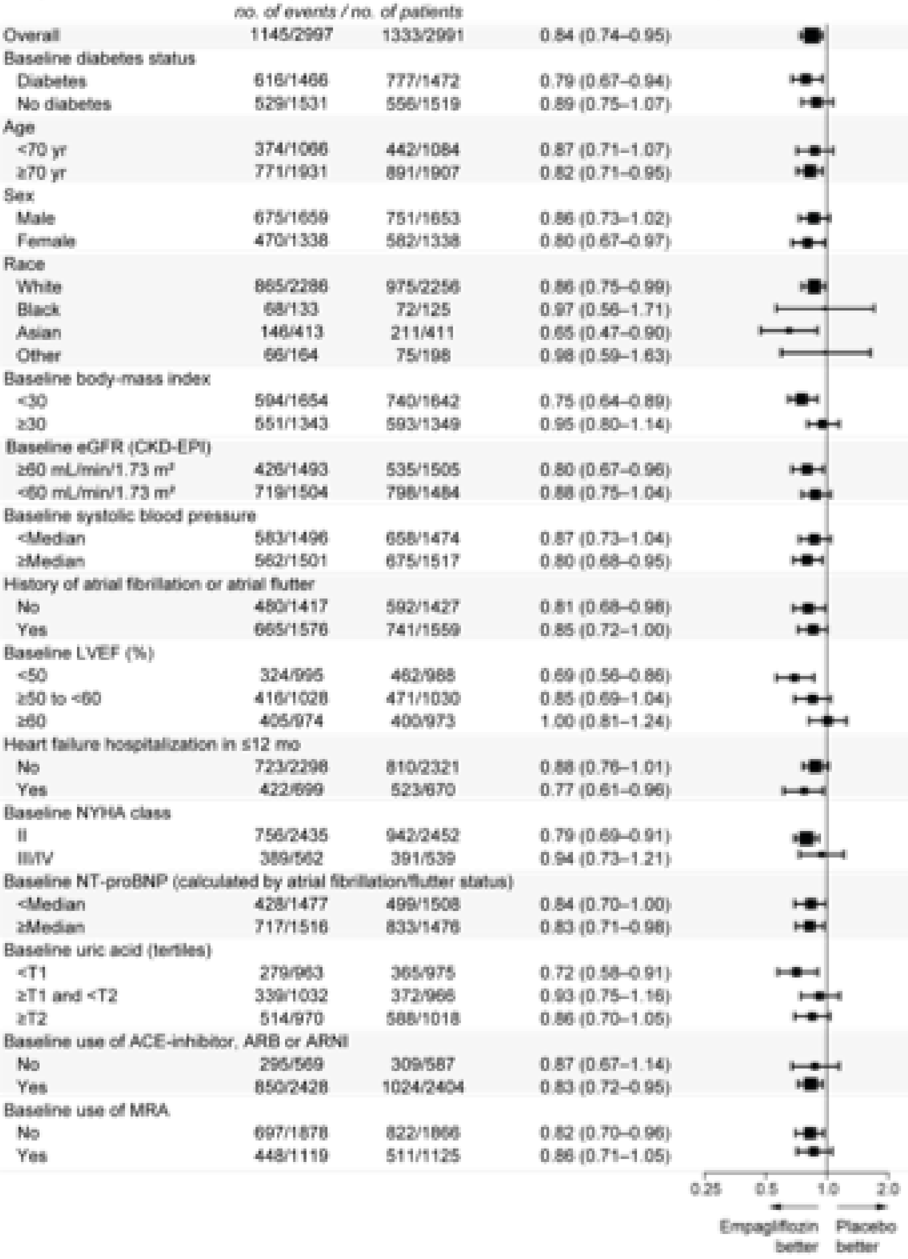

Trend test p-value is shown for baseline uric acid (using sex-specific tertiles, T1 and T2) and ejection fraction subgroups. The size of the squares for the hazard ratios is proportional to the size of the subgroup. The body-mass index is the weight in kilograms divided by the square of the height in meters. Race was reported by the patients. NYHA class II includes 4 patients with NYHA class I. Baseline uric acid tertiles calculated separately for males and females. ARNi= angiotensin receptor–neprilysin inhibitor, CKD-EPI= Chronic Kidney Disease Epidemiology Collaboration, HF= heart failure, eGFR= estimated glomerular filtration rate, LVEF= left ventricular ejection fraction, MRA= mineralocorticoid receptor antagonist, NT-proBNP= N-terminal prohormone of brain natriuretic peptide, NYHA= New York Heart Association.

Supplemental Figure III
Changes in hematocrit during double-blind treatment in the placebo and empagliflozin groups

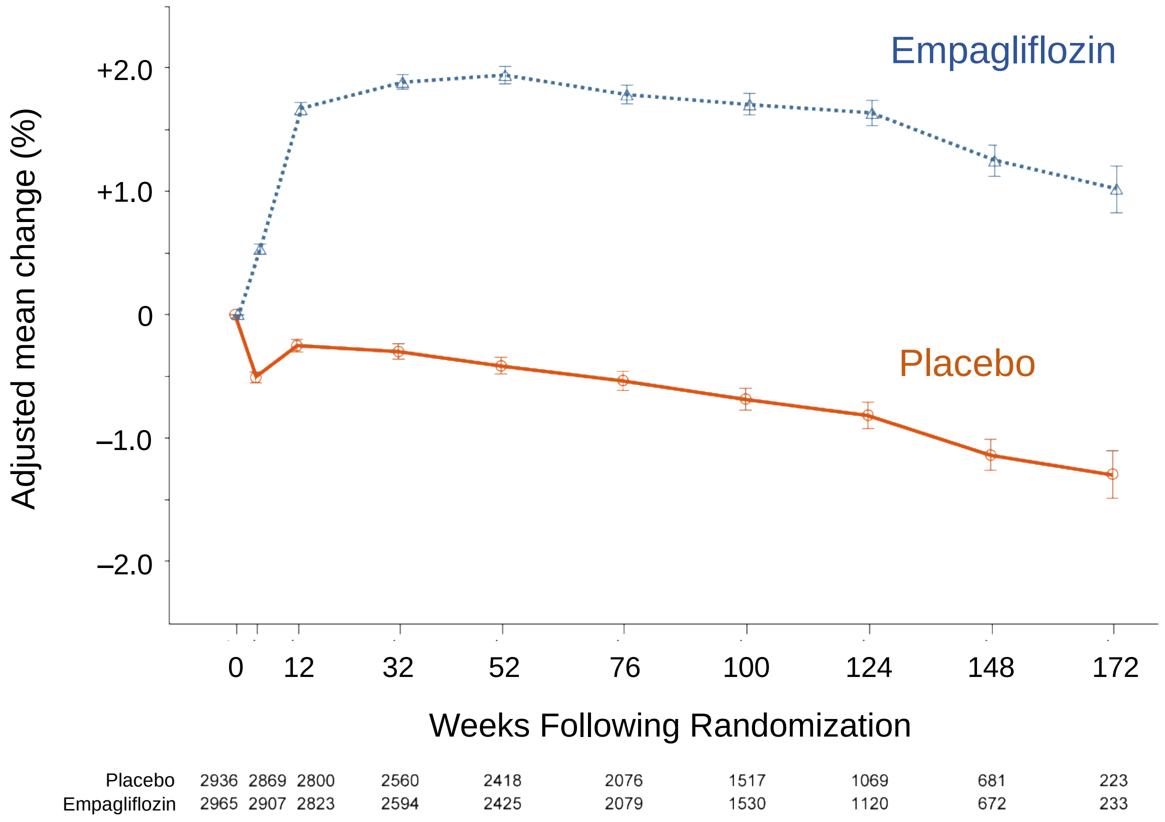

Shown are adjusted mean changes (and standard errors) at prespecified study visits based on mixed model for repeated measures, which included age and baseline estimated glomerular filtration rate and ejection fraction as linear covariates and baseline score by visit, visit by treatment, sex, region, individual last projected visit based on dates of randomization and trial closure, and baseline diabetes status as fixed effects.

Supplemental Figure IV

Changes in uric acid during double-blind treatment in the placebo and empagliflozin groups

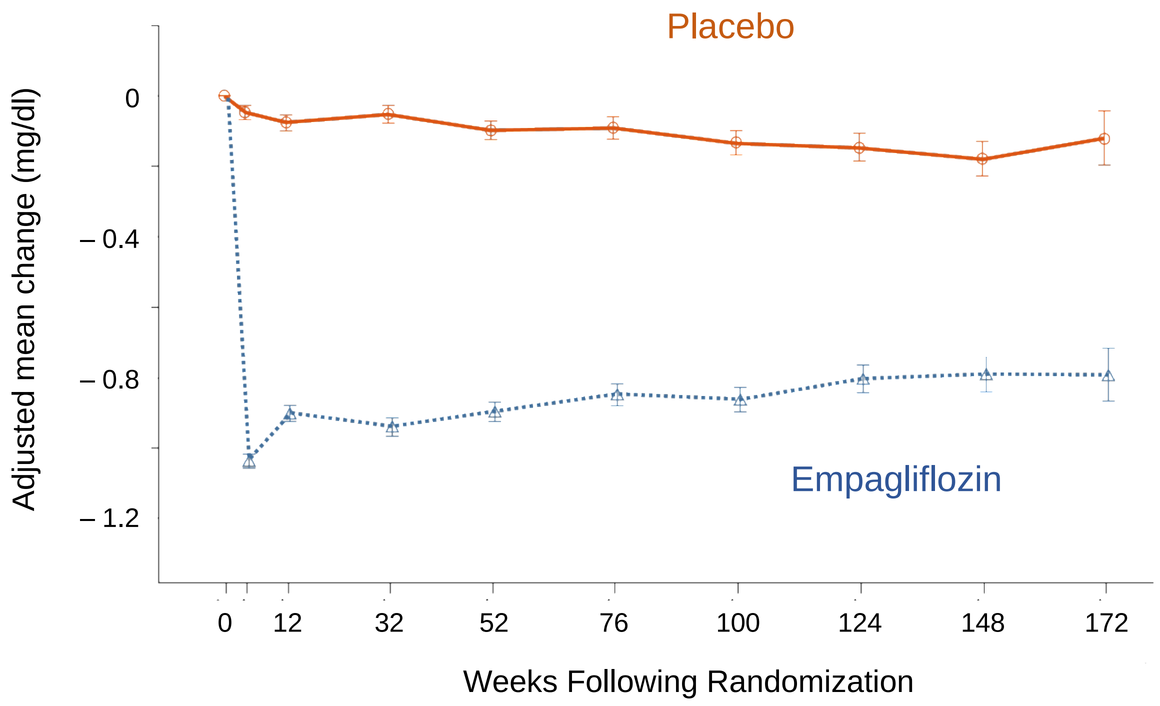

Placebo	2913	2881	2792	2567	2429	2099	1532	1086	691	230
Empagliflozin	2937	2901	2824	2604	2447	2092	1534	1116	680	241

Shown are adjusted mean changes (and standard errors) at prespecified study visits based on mixed model for repeated measures, which included age and baseline estimated glomerular filtration rate and ejection fraction as linear covariates and baseline score by visit, visit by treatment, sex, region, individual last projected visit based on dates of randomization and trial closure, and baseline diabetes status as fixed effects.

Supplemental Figure V

Changes in body weight during double-blind treatment in the placebo and empagliflozin groups

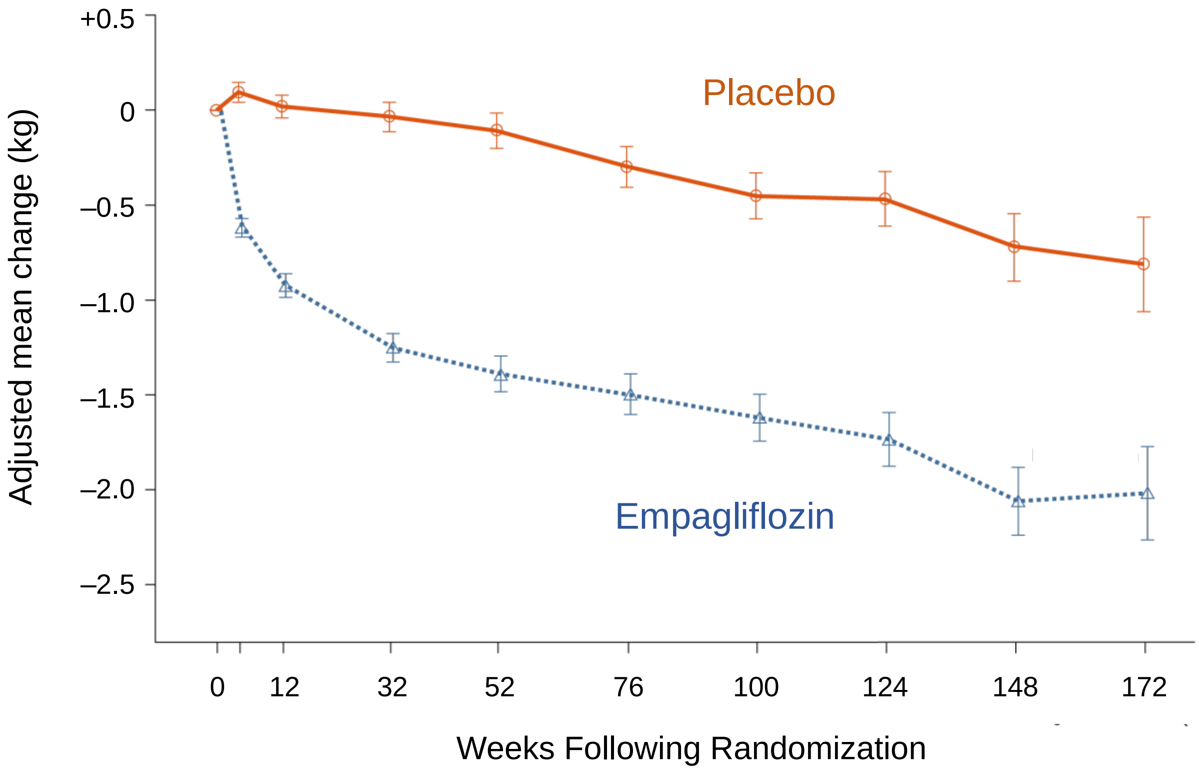

Placebo	2948	2925	2852	2636	2497	2169	1602	1127	704	239
Empagliflozin	2967	2951	2873	2662	2516	2172	1603	1154	704	247

Shown are adjusted mean changes (and standard errors) at prespecified study visits based on mixed model for repeated measures, which included age and baseline estimated glomerular filtration rate and ejection fraction as linear covariates and baseline score by visit, visit by treatment, sex, region, individual last projected visit based on dates of randomization and trial closure, and baseline diabetes status as fixed effects.

<u>Supplemental Figure VI</u>

Changes in N-terminal proBNP during double-blind treatment in the placebo and empagliflozin groups

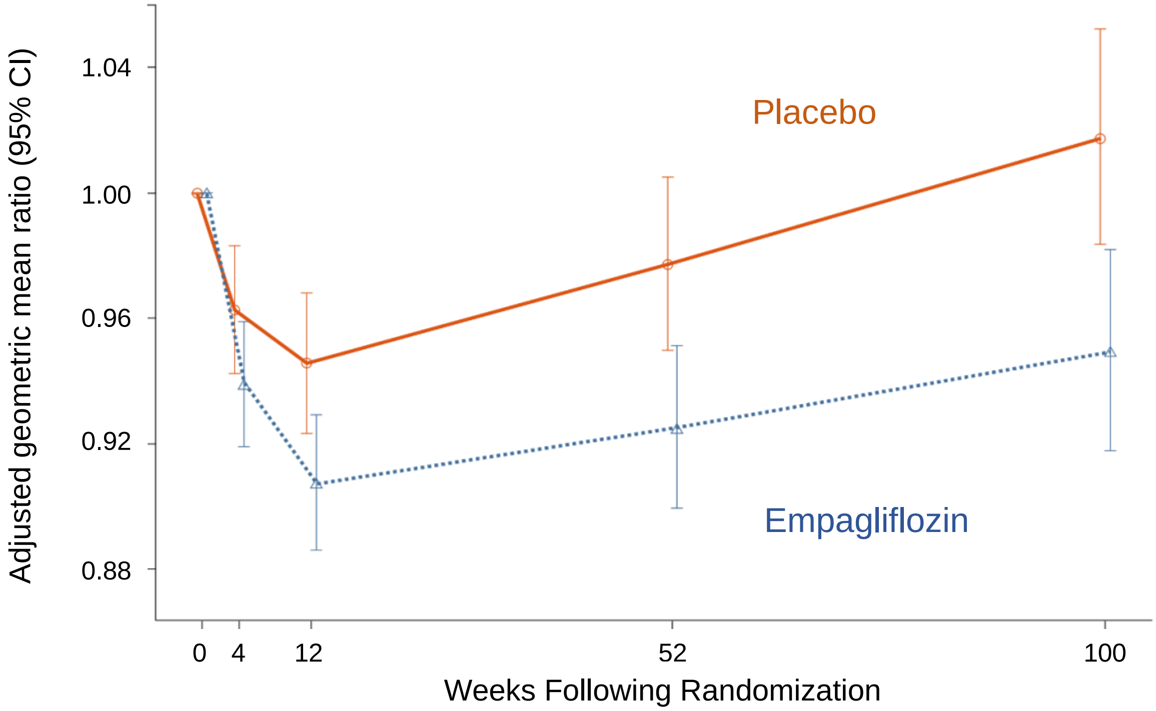

Placebo	2936	2884	2818	2460	1818
Empagliflozin	2964	2910	2846	2477	1814

Shown are adjusted geometric mean ratio and 95% confidence intervals at prespecified study visits based on mixed model for repeated measures, which included age and baseline estimated glomerular filtration rate and ejection fraction as linear covariates and baseline score by visit, visit by treatment, sex, region, individual last projected visit based on dates of randomization and trial closure, and baseline diabetes status as fixed effects. N-terminal proBNP was log-transformed prior the analysis.

Supplemental Figure VII

Changes in systolic blood pressure during double-blind treatment in the placebo and empagliflozin groups

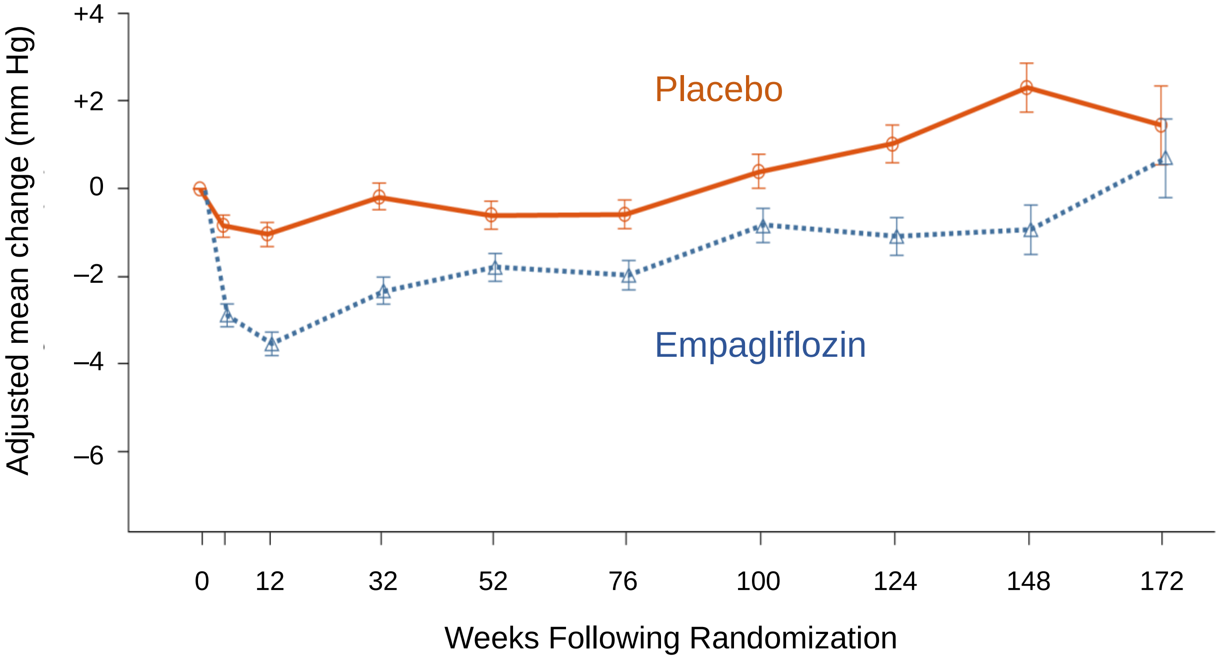

Placebo	2946	2925	2844	2619	2476	2147	1588	1112	702	241
Empagliflozin	2970	2955	2864	2645	2501	2145	1598	1145	702	247

Shown are adjusted mean changes (and standard errors) at prespecified study visits based on mixed model for repeated measures, which included age and baseline estimated glomerular filtration rate and ejection fraction as linear covariates and baseline score by visit, visit by treatment, sex, region, individual last projected visit based on dates of randomization and trial closure, and baseline diabetes status as fixed effects.
